# Supplementary material for: Reduced Cerebellar Volume in Term Infants with Complex Congenital Heart Disease: Correlation with Postnatal Growth Measurements
Source: Diagnostics (Basel). 2022 Jul 6;12(7):1644. doi: 10.3390/diagnostics12071644 (PMC9321214; doi:10.3390/diagnostics12071644)
Supplement: Supplementary file 1 [file diagnostics-12-01644-s001.zip › diagnostics-1778133-supplementary.pdf]

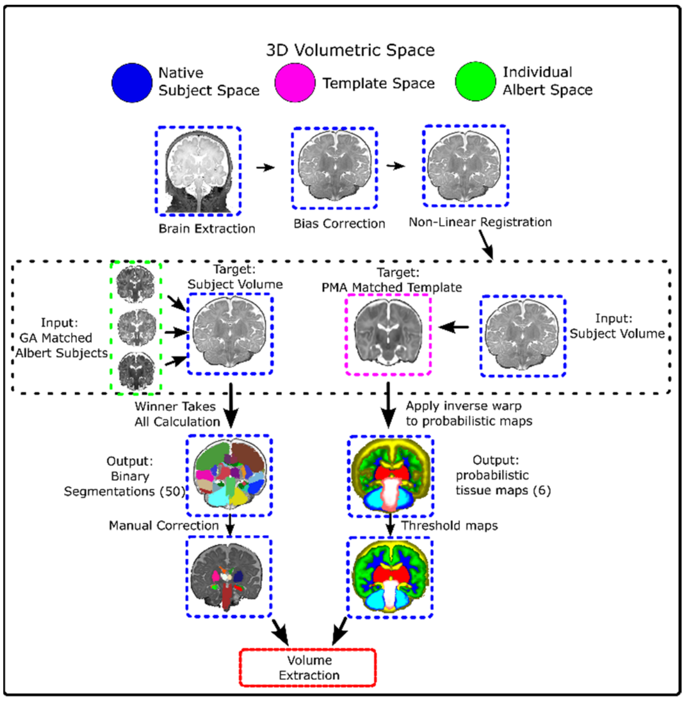

Figure S1. Semi-automated structural segmentation. We developed an automated structure segmentation pipeline (with a final manual correction step) that efficiently delineates 50 structures in native subject space. The only input required is a T2 weighted structural MRI.

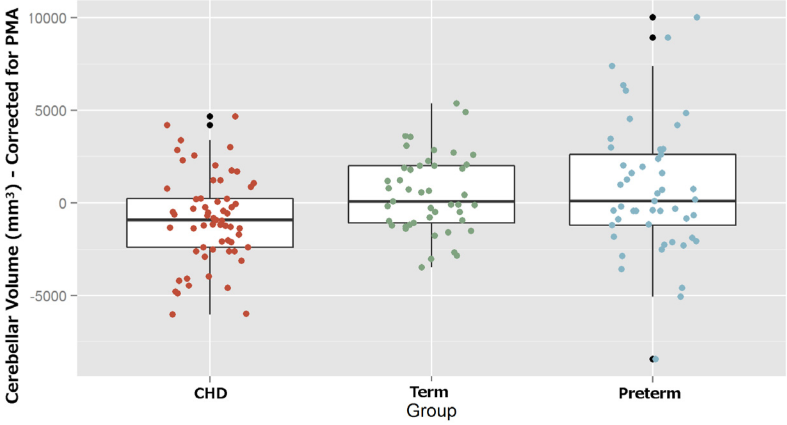

Figure S2. Cerebellar volumes (corrected for PMA) at the time of MRI. Volumes were corrected for post-menstrual age (PMA). CHD neonates showed a significantly lower volume when compared with both preterm born neonates and term born controls. No significant difference between the preterm and control groups was noted. ANOVA p-value < 0.002, with pairwise Tukey's HSD: control–preterm: p < 0.936; control–CHD: p < 0.015; preterm–CHD: p < 0.004.
